# Testing the extreme male hypothesis in the valproate mouse model; sex-specific effects on plasma testosterone levels and tyrosine hydroxylase expression in the anteroventral periventricular nucleus, but not on parental behavior

**DOI:** 10.3389/fnbeh.2023.1107226

**Published:** 2023-02-02

**Authors:** Neza Grgurevic

**Affiliations:** Institute of Preclinical Sciences, Veterinary Faculty, University of Ljubljana, Ljubljana, Slovenia

**Keywords:** valproate, sex difference, parental behavior, tyrosine hydroxylase, autism, testosterone, mouse

## Abstract

**Introduction:**

Autism is a neurodevelopmental disorder with a strong male bias in prevalence and severity. The extreme male hypothesis proposed that autism is a manifestation of extreme male traits as evidenced by increased masculine behaviors, hypermasculinization of some brain regions, and alterations in androgen metabolism. In the present study, the extreme male hypothesis was tested in the valproate (VPA) mouse model.

**Methods:**

Females of the C57BL/6JOlaHsd mouse strain were treated with 500 mg/kg VPA on gestational day 12. Offspring of both sexes were tested at 3 to 4 months of age in the elevated plus maze (EPM), open field, sociability tests, and for parental behavior. After sacrifice at 5 to 6 months of age, plasma testosterone was measured in males, while the brains of both sexes were examined for tyrosine hydroxylase (TH) expression in the anteroventral periventricular nucleus (AVPV).

**Results:**

VPA treatment significantly increased plasma testosterone levels and decreased AVPV TH expression in males, whereas the expression of TH in females remained at the same level. In parental behavior test none of the pup-oriented behavior was affected by VPA treatment in both sexes, the exception was nest quality which was lower after VPA exposure in males, but not in females.

**Discussion:**

Our results suggest a hypermasculinizing effect of VPA that occurred specifically in males but not in females, and this effect could be related to changes in androgen physiology. Nevertheless, a generalized interpretation of the extreme male hypothesis on brain and behavior should be avoided due to the complex effects of VPA.

## 1. Introduction

Autism is a neurodevelopmental disorder that encompasses a clinically heterogeneous group of disorders collectively known as autism spectrum disorders (ASD). A common feature of ASD is three behaviorally altered domains: social deficits, impaired language and communication, and stereotyped and repetitive behaviors (American Psychiatric Association, 2000). Due to a complex etiology in the majority of patients, the origin of autism remains unknown and represents an interaction of multiple genetic, environmental, and hormonal factors. One of the most consistent findings in ASD is the sex difference in prevalence and severity of the disorder. The ratio of male to female diagnoses has recently been estimated at 3:1 and could be as high as 11:1 in Asperger syndrome (Baron-Cohen et al., 2005; Loomes et al., 2017). The presence of a strong sexual dimorphism led to the extreme male hypothesis, which states that traits associated with the male sex are overemphasized, while typically female traits are diminished in autism. The effects of hypermasculinization in ASD patients could be seen in behavior and neuroanatomical features. ASD patients showed an extremely masculine profile in psychological tests, such as a strong drive to systemize and decrease ability to empathize (Baron-Cohen et al., 2011) and in terms of neuroanatomy, children with ASD have larger brains and amygdala than normal males (Baron-Cohen et al., 2005, 2011). The origin of hypermasculinization was proposed to be strongly connected with prenatal exposure to testosterone. Studies showed a positive correlation between fetal testosterone and non-social behavior, which is more common in males (Baron-Cohen et al., 2011). Geier and Geier (2007) measured increased androgen blood levels in boys with ASD, while Chakrabarti (Chakrabarti et al., 2009) showed alterations in androgen regulation and expression of genes involved in sex steroid metabolism in individuals with ASD. Valproic acid (VPA), a histone deacetylase inhibitor, is one of the drugs most commonly associated with increased ASD risk in humans. Due to behavioral and molecular parallels between VPA rodents and individuals with autism, prenatal exposure to VPA has been proposed as a valid animal model for autism (Rodier, 1996; Roullet et al., 2013; Tartaglione et al., 2019; Chaliha et al., 2020). Regarding sexual dimorphism, some VPA studies report male-specific changes at behavioral and molecular levels (Schneider et al., 2008; Hara et al., 2012, 2015; Kataoka et al., 2013; Kim et al., 2013, 2016; Kazlauskas et al., 2019), while others report that females are either more sensitive or exhibit different changes than those observed in males (Kazlauskas et al., 2016, 2019; Kim et al., 2016; Anshu et al., 2017; Melancia et al., 2018). In the VPA rodent model, adult social behavior has mostly been assessed using the three-chamber test or in a social interaction test with the same-sex animal [for a review, see Tartaglione et al. (2019), Chaliha et al. (2020)]. To our knowledge, there is no study focusing on a complex social behavior such as parental behavior. Studying parental behavior is valuable from two perspectives; first, it is one of the most sexually dimorphic behaviors in rodents, generally more pronounced in females than in males (Lonstein and De Vries, 2000), and second, it requires high social skills that are impaired in ASD individuals. Therefore, we questioned whether prenatal treatment with VPA might impair this behavior and whether this impairment is more pronounced in males than in females. Furthermore, it has been shown that thyrosine hydroxylase (TH)-expressing neurons in the anteroventral periventricular nucleus (AVPV) of the mouse hypothalamus are directly involved in parental behavior (Scott et al., 2015), and since this expression is sexually dimorphic in rodents (Simerly et al., 1985a,b), this marker was chosen to test for a possible hypermasculinization effect of prenatal VPA exposure. Based on evidence that ASD patients have elevated plasma androgen levels, we also measured plasma testosterone in VPA and control males. To control for other traits such as anxiety and interest in social stimuli, mice of both sexes were tested in the elevated plus maze (EPM), the open field fest (OFT), and the three-chamber sociability test, as these are the most commonly used tests in ASD research.

## 2. Materials and methods

### 2.1. Animals

All experimental animals were wild-type C57BL/6JOlaHsd mice bred in the Laboratory of Animal Genomics Veterinary Faculty, University of Ljubljana. Mice were housed under standard conditions: 12:12 dark-light cycle (LED lights, CCT 3000 k, not exceeding 25 lux above the cage) with temperature 21–24°C, humidity between 50–60%, and had irradiated rodent diet with minimal presence of phytoestrogens (Teklad global 16% protein rodent diet^®^, BN 2916, Envigo, Udine, Italy) and drinkable tap water with addition of 37% HCl (0.7 ml/L, Merck, Darmstadt, Germany; pH 3-4) to reduce the growth of microorganism. Food and water were available *ad libitum*. Mice were conventionally bred in open polycarbonate cages (Tecniplast, Buguggiate, Italy) on wood chip bedding (Lignocel^®^, Rettenmaier and Söhne, Germany). Six females, already experienced mothers, were mated to obtain prenatally VPA-treated test mice. Pregnancy was confirmed by the presence of a vaginal plug, which was considered embryonic day 0 (E0). Females with plugs were housed together. At E12, they were isolated in a single cage (13 cm × 28.5 cm × 10.5 cm) and treated with either VPA or vehicle (saline) as a control. VPA (sodium salt; Sigma) was dissolved in 0.9% saline and injected subcutaneously at a concentration of 500 mg/kg body mass in a volume of 0.01 ml. Experiments were performed on adult offspring prenatally exposed to VPA, a total of 20 females (10 control and 10 VPA) and 21 males (8 control and 13 VPA) from three different litters. At weaning, 21-day-old mice were separated by sex and housed in groups of 3–5 animals per cage (37.5 cm × 22 cm × 15 cm) during experiment. Animals from the same treatment were housed together. For determination of plasma testosterone, an additional 12 male animals were added (6 control and 6 VPA animals), which were not included in the behavioral tests and brain immunohistochemistry.

### 2.2. Behavioral tests

At 3–4 months of age, mice were tested during the light phase of a cycle in a battery of behavioral tests in the following order: elevated plus maze (EPM), open field test (OFT), sociability test, and test for parental behavior. In females, all tests were performed at the time of diestrus, which was confirmed by the vaginal smear (Caligioni, 2009). To provide similar handling conditions for both sexes, males were also consistently removed from the cage for three days prior to testing and including the day of testing. Like females, males were also gently held by the tail while sham genital smear was performed. Each mouse was tested in only one test per day, with at least three days between tests. Bedding was changed once per week, always at the end of the test. All tests were performed by the same person, who was blinded to the treatment.

#### 2.2.1. Elevated plus maze

The Elevated plus maze test was conducted in a black Plexiglas maze elevated 50 cm above the floor and consisting of two open and two closed arms, 66 cm long and 5 cm wide. A mouse was placed in the center of the maze and allowed to freely explore the maze. Each subject was tested for 5 min. The number of entries and the time spent in the open and closed arms were recorded. Behavior was assessed using the Stopwatch program (Center for Behavioral Neuroscience, Atlanta, GA, USA). Feces and urine were cleaned with 70% ethanol and the maze was dried until the next animal.

#### 2.2.2. Open field

The open field test was conducted in a square shaped maze (40 cm × 40 cm × 40 cm) made of dark Plexiglas and elevated 50 cm above the ground. A central zone of 24 cm × 24 cm was marked 8 cm from the wall. The mouse was placed in the central zone, and during the 30-min test, the number of crossings and the time spent in the central zone were recorded. Behavior was video recorded and analyzed using the ANY-maze software package (Stoelting Europe, Dublin, Ireland).

#### 2.2.3. Sociability test

The sociability test was conducted as described in an article by Nadler (Nadler et al., 2004). Transparent Plexiglas maze consisted of three chambers, each 20 cm long, 40.5 cm wide, and 22 cm high, separated by transparent Plexiglas walls with doors that could be opened or closed. The middle chamber served as the starting position to explore the other two chambers, which were used to stimulate social or non-social behavior. Social behavior was stimulated with an adult, sexually naive male of the non-aggressive A/J strain. During testing, stimulus mouse was placed in a wired cup with a height of 11 cm and a diameter of 10.5 cm. Bars were spaced 1 cm apart and allowed nasal contact between animals. All stimulus mice were habituated to the wired cups for several days prior to testing, and none of the test mice had any prior contact with the stimulus mice. The position (left or right chamber) of the cup was systematically changed between tests to avoid possible side preference. Each test consisted of three different stages. In the first, habituation stage, the test animal was allowed 5 min to freely explore an empty maze. In the second, 10-min stage, the test animal was given the choice of exploring a chamber with an empty cup or a chamber with a cup containing a stimulus animal (social cup). In the third (known-novel) stage, the empty cup was replaced by a cup containing a novel stimulus animal (novel cup). Again, the test animal was allowed to explore all three chambers for 10 min. The ANY maze program was used to assess several behavioral parameters: latency to enter the chambers, time spent in the chamber, time spent sniffing the cups, immobility. Between tests, the maze was cleaned with distilled water. Sociability index (SI) and Social Novelty Preference index (SNI) were evaluated using Baronio’s mathematical equation (Baronio et al., 2015):


SI=((timespentsocial-timespentempty)/(timespentsocial

+timespentempty))×100,SNI=((timespentnovel

-timespentknown)/(timespentnovel+timespentknown))×100

#### 2.2.4. Parental behavior test

One to two hours before testing, animals were isolated in a polycarbonate cage (36.5 × 20.7 × 14 cm) with fresh bedding and nesting material (Nestlet, Plexx, The Netherlands). Each mouse was allowed 1–2 h to habituate and build a nest. After habituation, three pups no more than seven days old of the same strain and of both sexes were added on the opposite side of the cage, furthest from the nesting material. A wreck with food and water was removed, the cage was covered with the lid and parental behavior was observed for 15 min. The second test was performed four days after the first test or at the next diestrus, in males and females. Mice were isolated in the same cage where the first test was performed. During the test, the following parameters were scored with Stopwatch program: latency to first visit pups, latency to retrieve pups into the nest, grooming the pups, crouching over the pups and nest building (latency, number of initiations and duration of each activity). Nest quality was scored from 0 to 3 (0.5–1 = nest material shredded, 1–2 = nest is present but not fully formed, 2–3 = complete nest, with bowl shaped sides and top).

### 2.3. Sacrifice, blood collection, and brain immunohistochemistry

At 5–6 months of age, mice were anesthetized with a mixture of ketamine (Vetoquinol Biowet, Gorzow, Poland; 100 μg/g BW), acepromazine (Fort Dodge Animal Health, Fort Dodge, IA, USA; 2 μg/g BW), and xylazine (Chanelle Pharmaceuticals Ltd., Loughrea, Ireland; 10 μg/g BW). All animals from the same cage were sacrificed on the same day, therefore females were randomly anesthetized in relation to ovarian cycle. By vaginal smear stage of ovarian cycle was confirmed. Four (2 VPA, 2 control) females were sacrificed in estrus, six (4 VPA, 2 control) in diestrus and six (3 VPA and 3 control) in metestrus. Mice were perfused with 4% paraformaldehyde (pH 7.4; Sigma) in 0.01 M phosphate buffer (PB; Sigma). Blood was collected from the left ventricle with a heparinized syringe and centrifuged at 3,000 rpm for 5 min at 4°C. Plasma was stored at −20°C for further analysis. Brains were collected and left overnight at 4°C in the same fixative and stored the next day in 0.01 PB at 4°C for subsequent immunohistochemistry. Immunocytochemistry was performed on floating brain sections as previously described (Grgurevic et al., 2012). Primary TH rabbit antibodies (Chemicon, Temecula, CA, USA, Cat#AB152) at the dilution of 1: 4,000 were used to detect brain TH expression.

### 2.4. Determination of plasma testosterone concentration

Plasma testosterone was measured using a commercial ELISA kit (Demeditec, Kiel-Wellsee, Germany, No. DEV 9911) according to the manufacturer’s instructions.

### 2.5. Data collection and analyzes of the brain

Brains that were sectioned at the wrong angle or damaged during immunohistochemistry were excluded from the study. The location of the AVPV was determined by the presence of the anterior commissure and third ventricle, as shown in the atlas of Paxinos and Franklin (2001). Digital images of brain regions were acquired using an Eclipse 80i microscope with a DS -Fi1 camera (Nikon, Tokyo, Japan) under 100x magnification. Only one section per animal was used to count TH immunopositive cells. The sections were counted twice independently by two observers, and the average number was used for further statistical analysis.

### 2.6. Statistical analysis

All results were analyzed using NCSS statistical software (Kaysville, UT, USA). Behavior measured with ANY maze was recorded and briefly checked that the program tracked the test animals correctly during the test. Two OFT and three sociability tests were manually re-scored with a Stopwatch, as the ANY maze software was unable to correctly track the test animal throughout the test. Only latency and duration were measured in these tests. In the sociability test, one VPA male spent the entire time in the middle chamber, so the index of sociability and the index of social novelty preference could not be calculated. In the parental behavior due to aggression, 9 of 82 tests were terminated earlier. Parameters in these tests were calculated to 15 min. Data distribution was tested using the Shapiro–Wilk test. Behavior in OFT and AVPV TH expression were analyzed using ANOVA with treatment and sex as factor variable. Sociability, EPM, parental behavior, and plasma testosterone data were not normally distributed, therefore Kruskal–Wallis ANOVA was used. Differences between groups were confirmed by Fisher’s LSD Multiple-Comparison test. All results are presented as mean ± SEM, and *p* < 0.05 was considered statistically significant.

## 3. Results

### 3.1. VPA treatment had no effect on behavior in the EPM neither in females nor in males

**FIGURE 1:**
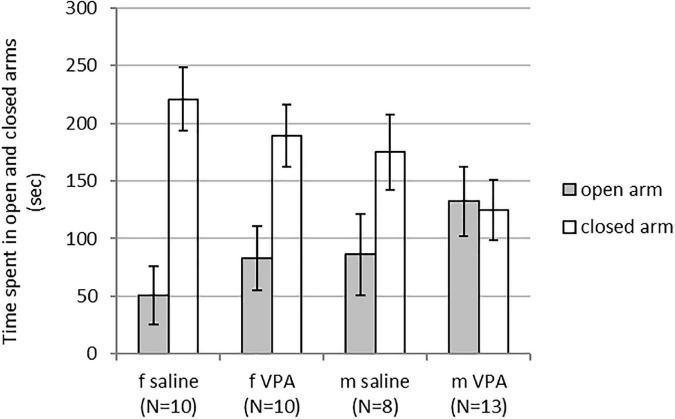
Behavior of control and valproate (VPA) mice in the elevated plus maze (EPM). No differences were observed between the groups.

Kruskal-Wallis ANOVA showed no statistically significant differences between groups. Time spent in the open [F(3, 40) = 1.43, *p* = 0.249] and closed arms [F(3, 40) = 2.29, *p* = 0.094] was similar in females and males of both treatments (Figure 1).

### 3.2. VPA treatment had no effect on behavior in the OFT, neither in females nor in males

**FIGURE 2:**
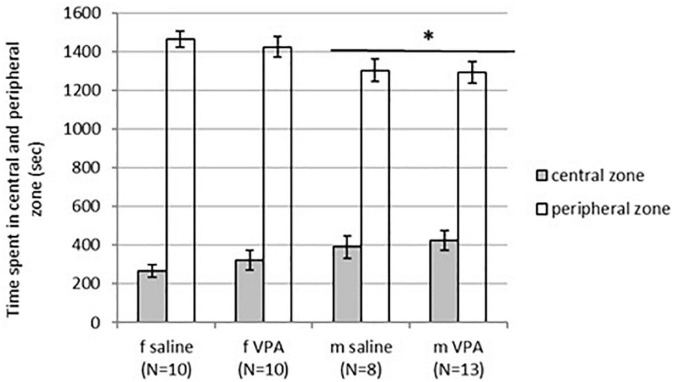
Behavior of control and valproate (VPA) mice in the OFT. Males spent statistically significantly more time in the central zone (p* < 0.05) and less time in the peripheral zone (p* < 0.05) compared to females. Differences between treatments were not significant.

No differences in behavior were observed between VPA and control mice in the OFT. Distance traveled [F(1,35) = 1.13, *p* = 0.295], line crossing [F(1,35) = 1.05, *p* = 0.311], and time of immobility [F(1,35) = 0.83, *p* = 0.367] were similar in both treatments. Balanced ANOVA showed sex differences in anxiety-like behavior. Males spent more time in the central zone [F(1, 37) = 4.96, *p* = 0.032] and less time in the peripheral zone [F(1, 37) = 6.89, *p* = 0.013] compared to females. Differences between treatments in time spent in the peripheral [F(1, 37) = 0.22, *p* = 0.644] as well as the central zone [F(1, 37) = 0.79, *p* = 0.378] were not significant (Figure 2).

### 3.3. The VPA treatment significantly lowered sociability index in males

**FIGURE 3:**
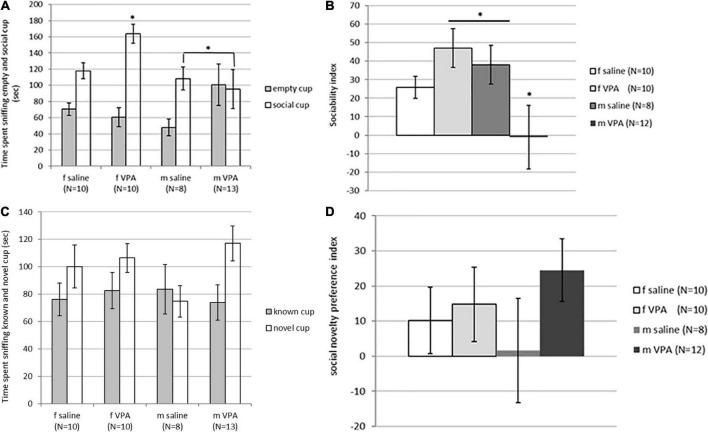
**(A)** Behavior of control and valproate (VPA) mice in the second stage of the sociability test. Mice were introduced with an empty (non-social) cup and a cup containing stimulus male (social). VPA females spent significantly more time sniffing the social cup than VPA males and control males (p* < 0.05). **(B)** Behavior of control and VPA mice in the second stage of the sociability test. Mice were introduced with an empty (non-social) cup and a cup containing stimulus male (social) cup. The sociability index was significantly lower in VPA males compared to saline males and VPA females (p* < 0.05). **(C)** Behavior of control and VPA mice in the third stage of the sociability test, in which the mice were introduced with a familiar (known cup) and novel stimulus animals (novel cup). No differences were observed between groups in time spent sniffing different cups. **(D)** Behavior of control and VPA mice in the third stage of the sociability test, in which the mice were introduced with a familiar (known cup) and novel stimulus animals (novel cup). No differences were observed between groups in the social novelty preference index.

Although time spent sniffing the non-social cup [F(3,40) = 1.62, *p* = 0.202] was not statistically significantly different between groups, time spent sniffing the social cup [F(3, 40) = 2.89, *p* = 0.048] reached statistical significance. VPA females spent significantly more time spent sniffing the social cup than males of both treatments (*p* > 0.05) (Figure 3A). The sociability index was also significantly different between groups [F(3, 39) = 2.96, *p* = 0.045]. VPA males had the lowest sociability index and were significantly different from saline males and VPA females (*p* > 0.05) (Figure 3B).

In the next stage of the test, when animals were challenged with a new stimulus mouse that replaced the empty cup, no differences were observed between groups. The time spent sniffing the new stimulus mouse [F(3, 40) = 1.74, *p* = 0.176], the time spent sniffing the familiar stimulus animal [F(3, 40) = 0.12, *p* = 0.946], and the social novelty preference index [F(3, 39) = 0.79, *p* = 0.510] did not differ between groups (Figures 3C, D). There were no differences between groups in immobility [F(3, 120) = 1.92, *p* = 0.129] and number of crossed lines [F(3, 120) = 0.44, *p* = 0.721].

### 3.4. The VPA treatment impaired nest quality in males only but had no effect on pup-oriented behavior neither in males nor in females

**FIGURE 4:**
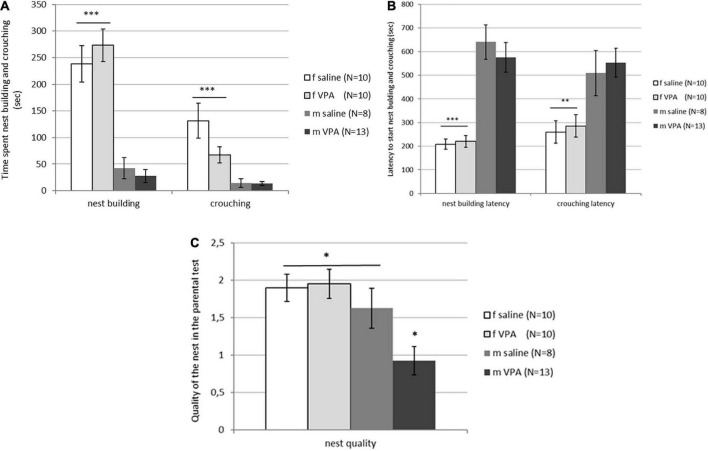
**(A)** Parental behavior of control and valproate (VPA) mice in the first and second tests. Females spent significantly more time crouching over the pups (p^***^ < 0.0001) and nest building (p^***^ < 0.0001) compared to males regardless of the treatment. **(B)** Parental behavior of control and VPA mice in the first and second tests. Latency to start crouching (p^**^ < 0.01) and nest building (p^***^ < 0.0001) was significantly shorter in females compared to males of both treatments. **(C)** Nest quality of control and VPA mice in the first and second test. VPA males possessed a significantly poorer nest than saline males and females of both treatments (p* < 0.05).

Typical sex differences were found in parental behavior. Females spent significantly more time crouching over the pups [F(3, 81) = 9.74, *p* = 0.00001] and nest building [F(3, 81) = 26.82, *p* = 0.00000] compared to males of both treatments (Figure 4A). Latency to initiate of crouching [F(3, 81) = 5.96, *p* = 0.0010] and nest building [F(3, 81) = 19.14, *p* = 0.00000] was also shorter in females compared to males despite treatment (Figure 4B). No differences in retrieving behavior were observed between treatments or between sexes. Only 6 of 20 females and 4 of 21 males retrieved at least one pup into the nest. In both sexes, half of the retrievers were VPA and the other half were control mice (data not shown). No differences in aggressive behavior were observed either. In the VPA males only 3 out of 13 and in the control males only 2 out of 8 were aggressive toward the pups, while in the females none of the animals were aggressive. Kruskal-Wallis ANOVA showed that nest quality was significantly different between groups [F(3, 81) = 6.16, *p* = 0.0008]. VPA males built poorer nests than any other three groups (*p* < 0.05) (Figure 4C).

### 3.5. The VPA treatment decreased the number of AVPV TH positive cells in males only

**FIGURE 5:**
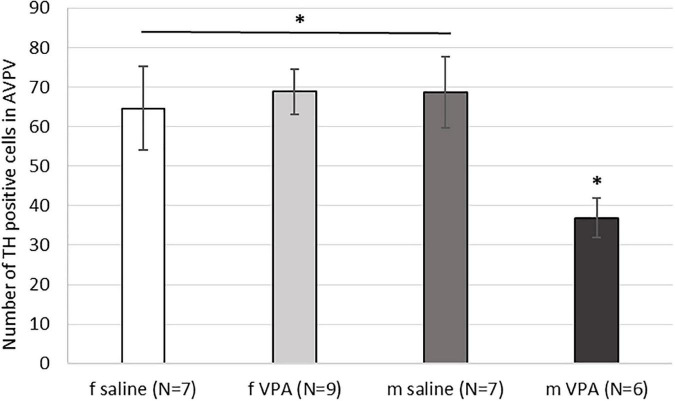
Expression of tyrosine hydroxylase (TH) immunopositive cells within the anteroventral periventricular nucleus (AVPV) in control and valproate (VPA) mice. VPA males had significantly lower (p* < 0.05) number of TH immunopositive cells than saline males and females of both treatments.

When the number of TH immunopositive cells in AVPV was analyzed, a statistically significant interaction [F(1, 28) = 5.15, *p* = 0.032] was found between sex and treatment. VPA males had significantly lower number of AVPV TH cells compared to saline males and females of both treatments (*p* < 0.05) (Figure 5).

### 3.6. Plasma testosterone was significantly elevated in VPA treated males compared to control males

**FIGURE 6:**
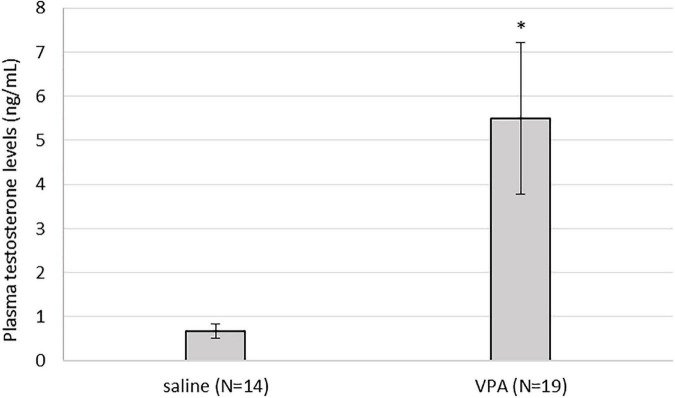
Plasma testosterone levels were statistically significantly higher in valproate (VPA) males compared to control males (p* < 0.05).

Kruskal-Wallis ANOVA showed that VPA males had a statistically significantly higher plasma testosterone level F(1, 32) = 5.76, *p* = 0.022 than control males, suggesting that prenatal VPA treatment increased plasma testosterone levels (Figure 6). The average number of AVPV TH positive cells in females sacrificed in three different ovarian cycles is shown in Table 1. Four (two VPA and two control) females were sacrificed in estrus, six (four VPA and two control) in diestrus, and six (three VPA and three control) in metestrus. Due to the small number of animals, data were not statistically analyzed (Table 1).

**TABLE 1:** Average number of anteroventral periventricular nucleus (AVPV) tyrosine hydroxylase (TH) positive cells in females sacrificed in three different ovarian cycle. Control and VPA females were pulled together.

Ovarian cycle in females (saline and VPA)	AVPV TH positive cells (mean ± SEM)
Oestrus (*N* = 4)	52.34 ± 2.70
Diestrus (*N* = 6)	79.13 ± 10.03
Metestrus (*N* = 6)	64.50 ± 8.51

## 4. Discussion

In the current study, we used the VPA mouse model to test the extreme male brain hypothesis in ASD. At the behavioral level, anxiety, sociability, and parental behavior were examined, while at the molecular level, brain expression of AVPV TH and plasma testosterone were measured. Our results revealed some novel aspects of the sex-specific effect of VPA treatment. Parameters such as sociability, nest quality, expression of AVPV TH and plasma testosterone levels were altered in a sex-specific manner, while anxiety-like behavior and parental behavior were not affected by VPA in either females or males. The influence of VPA on anxiety and sociability has been investigated in many studies. Some studies showed no effect of VPA treatment on sociability (Baronio et al., 2015; Eissa et al., 2018), while there are more studies showing decreased sociability index after VPA treatment (Kim et al., 2014; Guo et al., 2018), especially in males but not in females (Al Sagheer et al., 2018; Kazlauskas et al., 2019). Similar dicrepancies was found for anxiety. Kim et al. (2014) showed decrease in anxiety while several studies showed an increase in anxiety after VPA exposure (Kataoka et al., 2013; Lucchina and Depino, 2014; Gao et al., 2019). To compare all those results, one must carefully consider any differences between study designs, dose, route, and timing of VPA exposure, strain, or age at which the animals were tested (Tartaglione et al., 2019; Chaliha et al., 2020). For example, the recent study by Gao et al. (2019) reported excessive anxiety in VPA mice, but they used a dose of 600 mg/kg and reported a characteristic “kink” in the tail of VPA offspring, which is a sign of development impairment that could occur at a higher VPA dose (Schneider and Przewlocki, 2005). In our study, mice were exposed to 500 mg/kg VPA and showed none of the abnormalities such as tail deformity, body mass changes, or eye opening. Lucchina and Depino (2014) observed increased anxiety in mice at 600 mg/kg but not at 400 mg/kg VPA, while in the study by Kataoka et al. (2013), mice showed anxiety at 500 mg/kg VPA, which is in contrast to our study. Different mouse strains were used in all the above studies. Gao, Kataoka and their colleagues used CD -1 mice (Kataoka et al., 2013; Gao et al., 2019), while Lucchina and Depino used BALB/c × C57BL/6J hybrids (Lucchina and Depino, 2014) and C57BL/6JOlaHsd mice were used in our study. Since the strains differ in emotionality and cognitive performance (Wahlsten et al., 2003; Ennaceur, 2011), the use of a different strain could be a confounding factor. In addition, Anshu (Anshu et al., 2017), Kerr (Kerr et al., 2013) and their colleagues showed no effect on anxiety levels at 450 mg/kg or 600 mg/kg in rats.

Treatment with VPA had no effect on parental behavior in either females or males. As expected, behaviors such as crouching, and nest building were more prominent in females than in males regardless of treatment. Although VPA had no effect on pup-oriented behavior, it did affect nest quality, particularly in males, which built poorer nests compared with the other three groups. In agreement with our results, Kim et al. (2014) reported decreased nest quality, while Hill et al. (2015) found no changes in nest quality after VPA exposure. Hill et al. (2015) treated mice on gestation day 8.5 and used only males, while Kim et al. (2014) treated mice on gestation day 10 which is earlier than in our study. Although Kim and colleagues reported that all VPA-treated offspring were used in the study, sexes were not compared (Kim et al., 2014). Nesting is a complex behavior that, in addition to protective, camouflage, and thermoregulatory functions, is also a way to reduce stress and express play behavior. In other mouse models of ASD, such as Shank3 mice, authors describe the nest-building deficit as a result of increased novelty-induced anxiety and lack of interest in novel objects (Drapeau et al., 2018). We do not believe this was the case in our study, as no changes were observed in social novelty index and latency to first pup visit or latency to start nest building. The EPM and OFT results also show similar activity and anxiety levels. Therefore, the lower nest quality of VPA males in the current study may be due to impaired social behavior rather than anxiety or motor behavior. A similar explanation was given for Mecp2308/Y mice (Moretti et al., 2005), a model for Rett syndrome, although these mice also spent less time building nest which was not observed in our study.

Our results showed that treatment with VPA increased plasma testosterone levels and significantly decreased the expression of AVPV TH in males. A direct link between AVPV TH neurons and testosterone was described by Stephens et al. (2017). Selective ablation of TH in Kiss1 positive cells, which are located almost exclusively in the AVPV and are known to be master regulators of GnRH, indeed resulted in increased testosterone levels in male mice. Ovulation in females was not affected in the same study (Stephens et al., 2017). Similar, no changes in ovary cycling by VPA treatment were observed in our study. Because the expression of AVPV TH did not differ between VPA and control females, our results suggest that the hypermasculinizing effect of VPA at TH AVPV may be unique to males. At this point, it is not possible to determine whether the changes in the number of neurons from TH AVPV were secondary to increased plasma testosterone levels or whether testosterone levels were increased because decreased numbers of AVPV TH neurons. Male rodents are known to lose AVPV TH positive cells during prenatal development by testosterone-induced apoptosis (Simerly et al., 1985a,b). Therefore, it could be hypothesized that higher testosterone levels could lead to the loss of AVPV TH positive cells. However, in our study, testosterone was measured in adult males, which says nothing about prenatal testosterone. Nevertheless, gonadal hormones affect the expression of AVPAV TH even in adulthood. Simerly (1989) showed that additional treatment of gonadectomized rats with testosterone or estradiol decreased the expression of TH in AVPV, which is consistent with our results in which VPA males had higher testosterone levels and lower numbers of AVPV TH positive cells compared to control males. The same study could also explain the lack of sex differences in the expression of TH AVPV in our control animals. Because in our study the female animals were sacrificed at different ovarian cycles, we hypothesize that fluctuations in estrogen hormones could affect the expression of TH AVPV. Female animals sacrificed during estrus, when blood 17β-estradiol levels are highest (Wood et al., 2007), had lower expression of TH positive cells (52.34 ± 2.70) compared to diestrus (79.13 ± 10.03) and metestrus (64.50 ± 8.51) (Table 1). Although these results could not be confirmed with statistical significance, we speculate that the hormonal status of females may mask the sex difference that would otherwise occur in WT mice, but in VPA mice, increased testosterone may be the critical factor contributing to the sex difference in TH expression.

It appears that VPA acts on both systems TH brain expression and testosterone, but studies suggest that both effects are highly dependent on the timing of treatment. VPA administered to mice in adulthood did not alter TH protein levels or TH mRNA levels in the midbrain and striatum (Johnson et al., 2018), and similar results were shown by Liu et al. (2014) in the rat hippocampus. In contrast, a decrease in TH protein levels in the striatum was reported by Cezar et al. (2018) when VPA was administered prenatally to rats, by the same route as in us. A similar effect was observed for testosterone. When VPA was administered in adulthood, a decrease in blood testosterone was measured in humans (Ocek et al., 2018) and rats (Sukhorum and Iamsaard, 2017), which is in contrast to our results where VPA was administered prenatally.

In addition to the influence of VPA on the endocrine system and TH expression, VPA could directly influence sexual differentiation through its epigenetic effects. In two studies by Murray, the drug was injected into newborn mice during the critical period of sexual differentiation, and two sexually dimorphic brain regions were examined. In the first study (Murray et al., 2009), demasculinization of the bed nucleus of stria terminalis (BNST) was observed, while in the second study (Murray et al., 2011), the opposite effect was observed in the number of vasopressin fibers. Neonatal VPA treatment resulted in a masculinization effect in vasopressin fibers of the lateral septum in females, while in other brain regions the same fibers were increased in both sexes, suggesting a hypermasculinization or masculinization effect in males and females, respectively. Interestingly, in the same study, testosterone and estradiol levels were also measured in gonadally intact mice and no effect of VPA was found (Murray et al., 2011). In both studies (Murray et al., 2009, 2011), VPA was injected neonatally, while in our study VPA was injected prenatally, so again the timing of VPA application could be one of the important factors affecting the outcome of the study.

Taken together, it appears that VPA has a diverse and far-reaching influence on various aspects of the organism: Brain, behavior, and physiology, and interacts with sexual dimorphism. The present study was the first to investigate parental behavior and AVPV TH expression in the adult brain in the VPA mouse model. We showed that VPA had no effect on parental behavior, but affected sociability, nest quality, and expression of TH in the AVPV and increased plasma testosterone levels in males. Our results suggest a hypermasculinization effect in males, particularly on the expression of TH in the AVPV and testosterone levels. However, because VPA regulates numerous genes and its effect highly dependent on many different factors, all VPA studies should be interpreted with caution, and the hypermasculinization effect should not be readily extrapolated to brain and behavioral systems other than those that are the subject of the particular study.

## Data availability statement

The original contributions presented in this study are included in this article/supplementary material, further inquiries can be directed to the corresponding author.

## Ethics statement

The animal study was reviewed and approved by Administration of the Republic of Slovenia for Food safety, Veterinary and Plant protection; license number 34401-32/2012/8.

## Author contributions

NG designed the study and performed behavioral tests, brain and testosterone analysis, and statistical analysis.
